# Survey of international pediatric nutritional supportive care practices: a report from the Pediatric Study Group of the Multinational Association of Supportive Care in Cancer (MASCC)

**DOI:** 10.1007/s00520-024-08826-3

**Published:** 2024-09-07

**Authors:** Charles A. Phillips, Regina Kennelly, Catherine Carroll, Faith Gibson, Caitlin W. Elgarten, Andrea Orsey, Jason L. Freedman

**Affiliations:** 1 Division of Oncology, Children’s Hospital of Philadelphia, 3500 Civic Center Boulevard, Philadelphia, PA 19104 USA; 2 Department of Pediatrics, Perelman School of Medicine, University of Pennsylvania, Philadelphia, PA USA; 3 Department of Biomedical and Health Informatics, Children’s Hospital of Philadelphia, Philadelphia, PA USA; 4 Pediatrics Residency Program, Children’s Hospital of Philadelphia, Philadelphia, PA USA; 5 National Children’s Cancer Service, Children’s Health Ireland, Dublin, Ireland; 6 School of Health Sciences, University of Surrey, Guildford, UK; 7 Centre for Outcomes and Experience Research in Children’s Health, Illness and Disability, Great Ormond Street Hospital for Children NHS Foundation Trust, London, UK; 8 Center for Cancer and Blood Disorders, Connecticut Children’s Medical Center, Hartford, CT USA; 9 Department of Pediatrics, University of Connecticut School of Medicine, Farmington, CT USA

**Keywords:** Pediatric oncology, Malnutrition, Undernutrition; Clinical nutrition; malnutrition; Pediatric oncology

## Abstract

**Purpose:**

Malnutrition is common in children with cancer. While multiple validated malnutrition screens exist, there is no universal, standardized approach to screen or diagnose malnutrition. The Multinational Association of Supportive Care in Cancer (MASCC) Pediatric Study Group is focused on oncologic supportive care for children and young adults. This subgroup designed and administered a pilot study to characterize global malnutrition screening, diagnosis, and treatment practices for pediatric patients with cancer after identifying variations in malnutrition practice patterns within its members.

**Methods:**

A novel, exploratory survey was iteratively developed and distributed in early 2020 to 45 MASCC Pediatric Study Group members. The survey included multiple questions with standard patient presentations and nutritional scenarios, and the respondents selected the answer that best reflected the care patients would receive at their institution.

**Results:**

A validated screening tool to assess for malnutrition was routinely used by 15 of 26 respondents (58%). No single validated screen was used by more than 24% of responders, and 11 of 26 (42%) reported not having a standard malnutrition treatment screen. When the same patient was presented with the survey using different malnutrition indicators, patient care plans varied greatly. This was particularly true for z-scores compared to weight percentiles.

**Conclusions:**

Development of consensus recommendations for screening practices, preferred malnutrition indicators, and treatment guidelines could help reduce the underdiagnosis of malnutrition and subsequent variation in its management and ought to be a focus of the global pediatric cancer supportive care community.

**Supplementary Information:**

The online version contains supplementary material available at 10.1007/s00520-024-08826-3.

## Introduction

Malnutrition related to undernutrition is common in pediatric patients with cancer with a prevalence that varies by cancer type and is as high as 65% [1–5]. Recognition of malnutrition is critical as it negatively impacts physical and cognitive development, wound healing, immune function, mortality, and quality of life [3, 6–8]. Current recommendations from the United States Academy of Nutrition and Dietetics are for all hospitalized children to be screened for nutritional status [9, 10]. Nutrition screens assess variable combinations of dietary intake, anthropometric values, comorbid disease states, and subjective assessments of body habitus [10, 11]. While multiple validated screens exist, yet there is no universal, standardized approach to screening pediatric inpatients [9–15]. Without a uniform standardized screening tool to assess for malnutrition in patients, healthcare providers used a wide variety of approaches to diagnose and treat these children. This leads to discrepancies in the care of this population, which may cause poorer outcomes. In addition, this inconsistency leads to a lack of reliability and reproducibility when trying to compare nutritional status among and between patients and makes nutritional outcomes more difficult to assess longitudinally.

Publication of the indicators recommended for identification and documentation of pediatric malnutrition was released in 2014 by the Academy of Nutrition and Dietetics (AND) and the American Society for Parenteral and Enteral Nutrition (ASPEN) [16, 17]. The pediatric recommendations include the evaluation of z-scores for anthropometrics, weight trends, and nutritional intake. These differ slightly from the Royal College of Nursing guidelines which include BMI score, weight loss score, and acute disease effect score [18]. It is important to note that there is no one-size-fits-all approach; resources to diagnose malnutrition vary greatly by country, especially between high-income countries and low- and middle-income countries [19, 20]. In one study based out of pediatric hospitals in Nicaragua and Honduras, the providers used BMI for hematologic malignancies and MUAC (mid-upper arm circumference) for solid tumors. The use of these malnutrition indicators was based on the WHO guidelines for pediatric patients with cancer [21].

The Multinational Association of Supportive Care in Cancer (MASCC) is an international, interdisciplinary organization dedicated to research and education in all aspects of supportive care for people with cancer (www.mascc.org). The MASCC Pediatric Study Group is focused on oncology-supportive care for children, adolescents, and young adults. Its members include dietitians, nurses, pharmacists, and physicians. This subgroup designed and administered this pilot study after multiple meetings identified malnutrition practice pattern variations as a potential area for exploration.

Given the importance of diagnosing and treating malnutrition as well as the lack of uniformity in diagnosis and screening recommendations, this pilot study sought to leverage an international pediatric oncology supportive care group to better understand practice patterns for nutritional supportive care to pediatric cancer patients around the globe and examine approaches to malnutrition treatment for standardized patients. This information is critically important as it can provide insights into the shared points of emphasis, gaps in practice, and variability among providers that may need to be targeted for future international consensus guidelines.

## Methods

To better characterize global malnutrition screening, diagnosis, and treatment practices for pediatric patients with cancer, a survey (see Appendix) was developed iteratively by members of the MASCC Pediatrics Study Group in 2019 and 2020. It was distributed in early 2020 to 45 MASCC Pediatrics Study Group members. Surveys were distributed via email and results were collected via SurveyMonkey (www.surveymonkey.com). If the MASCC member felt they did not have the necessary experience or knowledge to complete the survey accurately, they were allowed to forward it to another person at their institution. The survey captured the respondents’ demographics, practice setting, and their approach to screening, diagnosis, and treatment of malnutrition. Using standardized patient scenarios, the respondents were asked to select the answer choice that most accurately reflected the care that patients would receive at their institution. Given varied criteria exist with no determined gold standard, the survey intentionally included questions that framed the nutritional scenario of the same patient in different ways, once using z-score-based framing and once using weight-centile–based framing to assess what respondents would choose with regard to diagnosis and treatment of malnutrition based on slight differences in how the patient was presented (To see the survey in its entirety, please see the Appendix.). The survey was approved by the Institutional Review Board at Children’s Hospital of Philadelphia and deemed exempt from full review. Descriptive parameters were reported for the demographic items. Respondent counts were characterized for the clinical care practice and standardized patient questions.

## Results

**Fig. 1:**
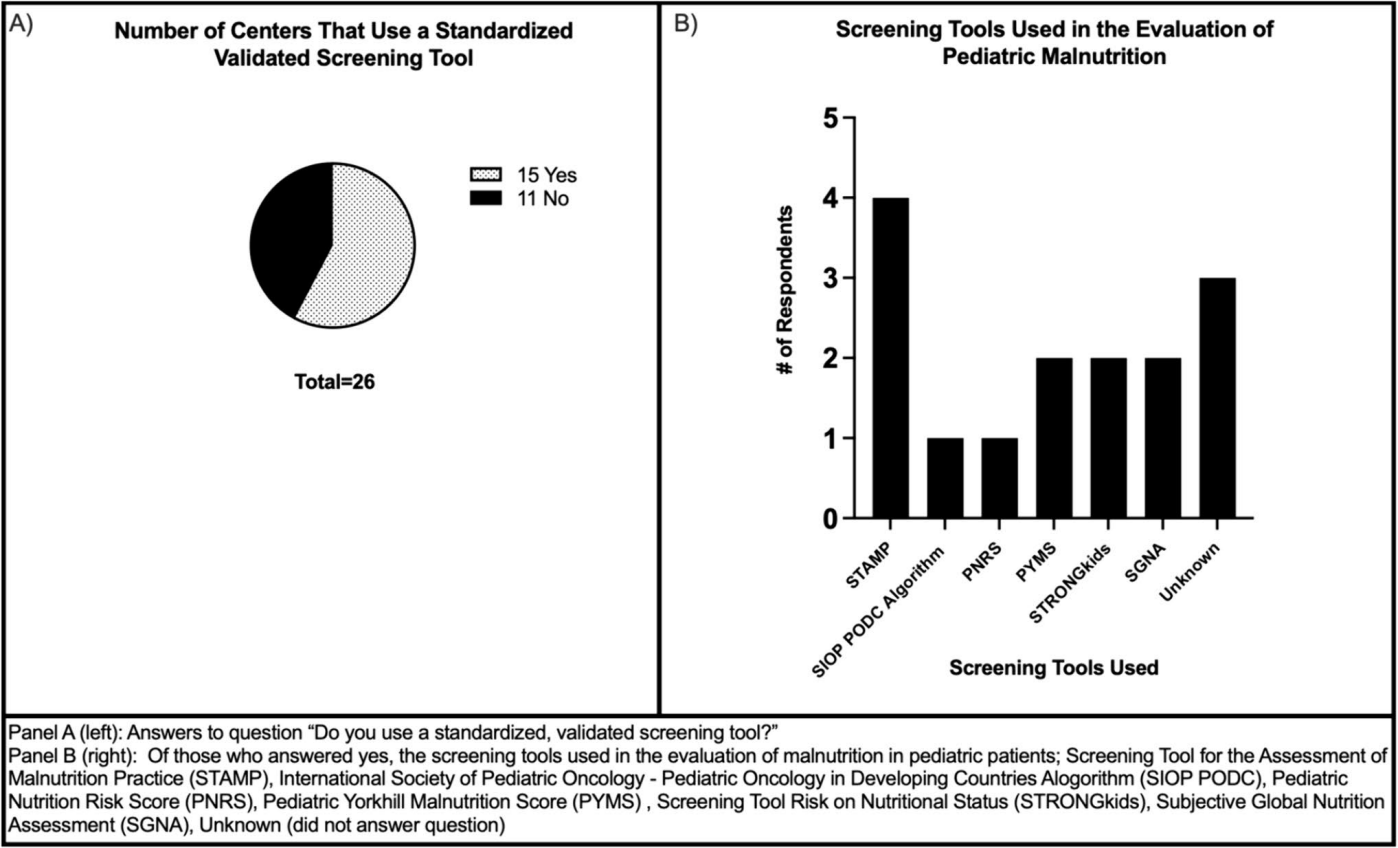
Malnutrition screening tools

Of the 45 members of the MASCC Pediatrics Study Group members who received the survey, complete responses were obtained from 19 respondents with an additional seven respondents who partially completed the survey but omitted at least one response. Responses came from 20 institutions across 11 countries with multiple members of the care team completing the survey including physicians, nurse practitioners, and registered dietitians (Table 1). When asked if the institution used a standardized, validated screening tool to assess for malnutrition, 15 of 26 survey respondents said “yes” (57%) (Fig. 1). Of those 15 who said yes, there was a wide variety of screening tools used. STAMP (Screening Tool for the Assessment of Malnutrition Practice) was the most utilized and was reported by four participants. Furthermore, 11 responders (42%) reported not having a standard malnutrition treatment guideline at their institution.

**Table 1:** Survey respondents by location, center, and discipline

Location	Name of center	Discipline of respondents (*n*)	
		Medical doctor (MD) or nurse practitioner (CRNP)	Registered dietician (RD)
Argentina	Hospital Garrahan	1	
Australia	Monash Children’s Hospital	1	1
Canada	SickKids Hospital		1
England	Leeds Children’s Hospital		1
	Royal Marsden NHS Trust		1
	Royal Manchester Children’s Hospital		1
	Bristol Royal Children’s Hospital		1
India	Tata Memorial Centre	1	
Ireland	Children’s Health Ireland		2
New Zealand	Starship Children’s Hospital		1
Romania	Oncology Institution Bucharest	1	
Russia	First Pavlov State Medical University of Saint Petersburg	1	
Scotland	NHS Greater Glasgow and Clyde		1
	Royal Hospital for Sick Children		1
United States	Cook Children’s Medical Center	1	
	Children’s Health of Orange County	2	
	Children’s Hospital of Philadelphia	2	1
	Memorial Sloan Kettering Cancer Center		1
	Connecticut Children’s Medical Center	1	1
	McMaster Children’s Hospital		1
Total	20 hospitals in 11 countries	11	15

**Fig. 2:**
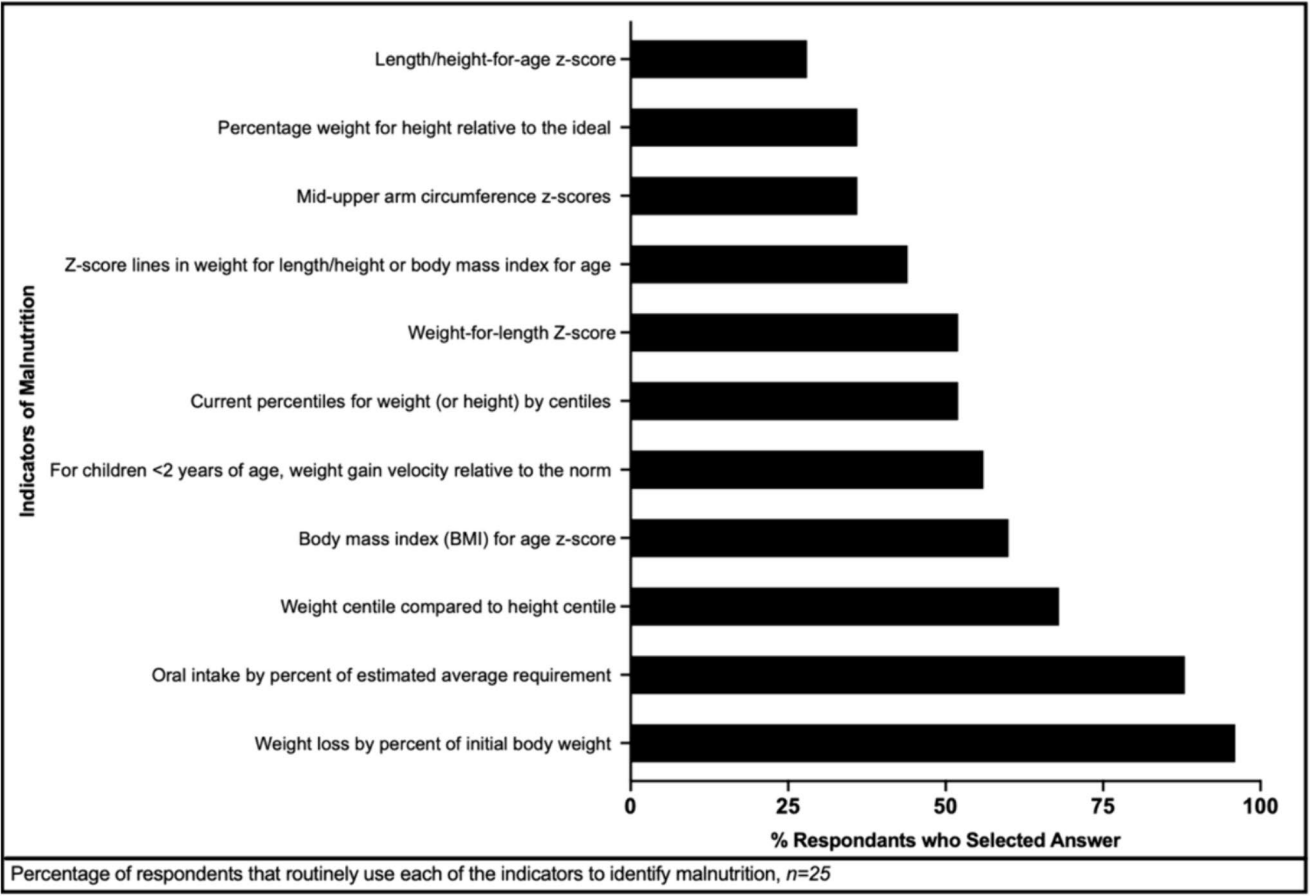
Malnutrition indicators in routine use

Respondents were asked to identify indicators of malnutrition used at their institution (Fig. 2). Of the 25 people who responded to this question, the most used indicator was weight loss by percent of initial body weight (24/25, 96%), followed by oral intake by percent of estimated average energy requirement (22/25, 88%). No other indicator was used by at least 70% of the respondents. The indicator utilized the least was length/height-for-age z-score at 28%.

During the standardized patient section of the survey, respondents had varied degrees of agreement with the diagnoses and suggested nutrition plans across multiple patient scenarios and various diagnoses. When the scenario presented a patient with newly diagnosed Stage 2 Wilms’ tumor with BMI z-score = 0 (normal weight), approximately half (12/22, 55%) responded that they would perform a nutrition screen and consult nutrition. Eight out of 22 (36%) said they would perform the nutrition screen but not consult nutrition, and 1/22 (5%) said they would do neither. In the same patient with a BMI z-score =  − 2.2, the majority (19/22, 86%) said they would perform a nutrition screen and consult nutrition.

The next scenario presented a patient with newly diagnosed high-risk acute myeloid leukemia (AML) with a plan for future transplant at BMI z-score = 0. When compared to the patient with Wilms’ tumor with the same z-score, a higher percentage would do a nutrition screen and consult nutrition at (17/22, 77%). For patients with AML with a BMI z-score of − 2.2, most survey takers (20/22, 91%) would screen and consult nutrition.

**Fig. 3:**
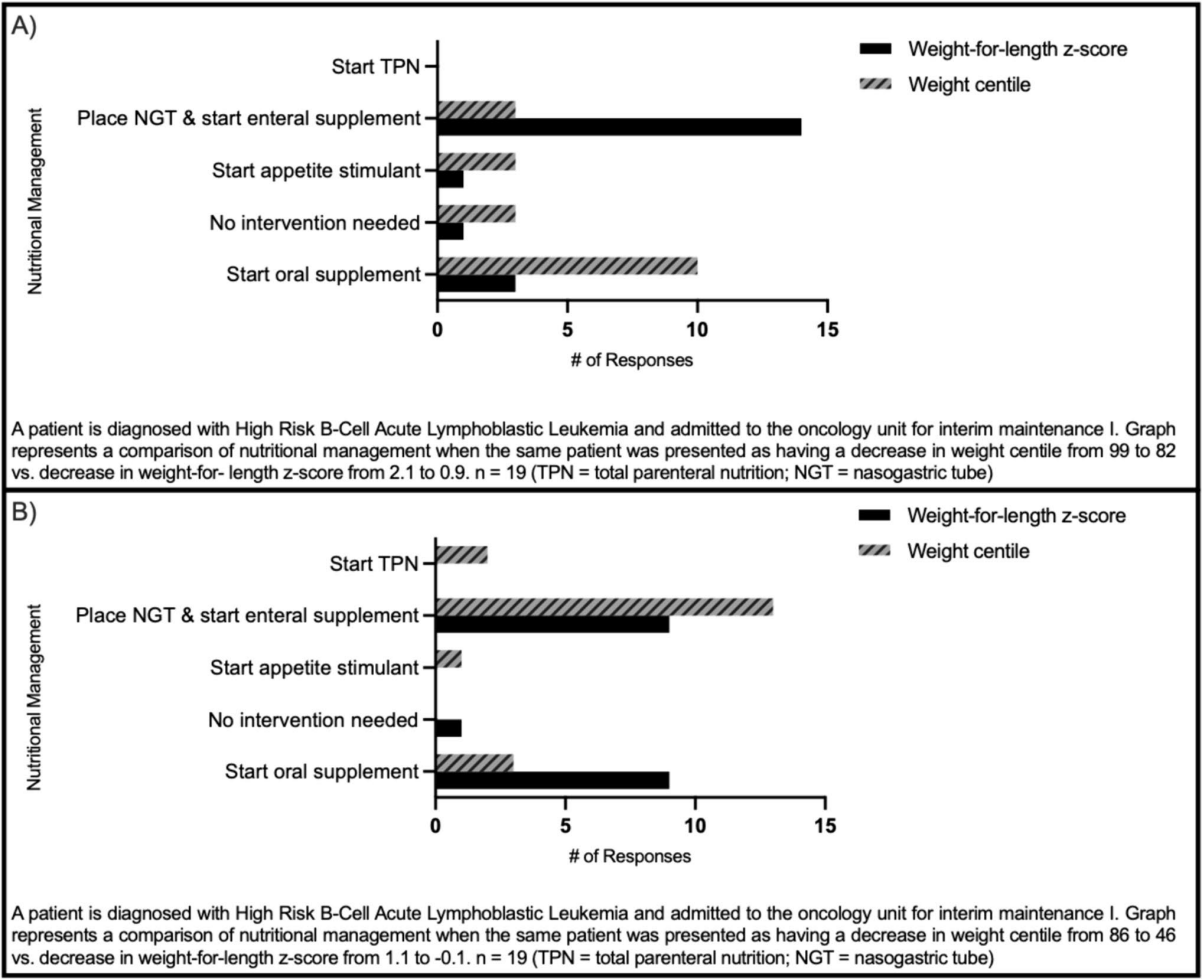
Nutritional management of HR B-ALL patient with decreasing z-score vs. weight centile

The last part of the survey presented a scenario of a patient with newly diagnosed high-risk B-acute lymphoblastic leukemia (ALL) using different indicators of malnutrition. The patient was presented with a decrease in weight centile followed by the corresponding weight-for-length z-scores. When the patient was presented as having a decrease in weight centile from 99 to 82, half of the respondents would start an oral nutritional supplement (10/19, 52%) (Fig. 3). If the same patient was presented with a drop in weight-for-length z-score from 2.1 to 0.9, most survey takers would place a nasogastric tube (NGT) and start enteral feeding (14/19, 74%).

Similar discrepant results were seen when the standardized patient was presented as having decreased in weight centile from the 86th percentile to the 46th percentile compared to a z-score of 1.1 to − 0.1. In this case, for the weight centile presentation, 13/19 (68%) would place an NGT tube and start enteral feeds compared to 9/19 (47%) when presented as z-scores.

## Discussion

This study surveyed a small but representative sample of providers as part of the MASCC Pediatric Study Group who care for pediatric oncology patients across the globe. First, it is notable that less than 60% of centers are performing nutritional screening as part of their clinical operations. Secondly, there is wide variability in approach to nutritional screening including which tools to use and when and how often these screens ought to be performed.

These results are consistent with what is felt globally; there is notable heterogenicity among professional societies about the parameters that should be used to diagnose and/or treat malnutrition. For example, the Royal College of Nursing 2014 Nutrition Guideline recommends starting enteral nutrition for weight two centiles below the height centile. However, the Consensus Statement by the Academy of Nutrition and Dietetics/American Society for Parenteral and Enteral Nutrition, also released in 2014, did not include weight being two centiles below height as a malnutrition indicator. Instead, it included z-scores for weight-for-height, length/height-for-age, body mass index (BMI) for age, and mid-upper arm circumference measurements. The Royal College of Nursing Nutrition Guidelines did not include any of these malnutrition indicators. To that end, here we see respondents showing limited to no consensus on which malnutrition indicators they favor. While the vast majority of respondents (96%) used weight loss by percent of initial body weight, closely followed by (88%) assessment of oral intake by percent of estimated average energy requirements, nine different indicators were also used in clinical practice including (length-for-age z-score (28%) and weight centile compared to height centile (68%) of the time.

Given this variation, an initial step for global pediatric oncology nutritional supportive care could entail ensuring malnutrition screens, whichever one(s) a center is most familiar with and is using standardly, are performed at diagnosis and at regular time points thereafter systematically. Perhaps the repeated nature of these assessments would demonstrate concerning trends in nutritional status if performed more routinely across the cancer experience. Furthermore, due to the numerous screens in use and relatively low screening rate, we would encourage pragmatism and support using a screen that does not require a significant amount of resources or time. Beyond the validated screens, at least one institution has used an automated, electronic screen [9, 22]. Efforts to validate and incorporate screens requiring the fewest resources should be prioritized so they are more easily integrated with minimal technology, effort, or cost. Additionally, with at least 11 different malnutrition indicators in use over 25% of the time, focus should be made on the areas of agreement, most notably weight loss by percent of initial body weight and oral intake by percent of estimated average energy requirement. Whichever screens are used, regardless of how screens are completed, using common indicators of malnutrition would enable better comparison of patients and nutrition-related outcomes globally.

Another significant area of heterogeneity was observed in which nutrition care plan respondents would recommend for a particular patient. While each scenario had some variability in responses, in general, the recommended nutrition support escalated in a stepwise fashion within cancers and across different diagnoses. For example, more respondents would start enteral nutrition via an NGT for a patient with Wilms’ tumor or AML with a z-score of − 2.2 but not for a patient with the same diagnoses and a z-score of 0. Additionally, more respondents would start nasogastric tube feeds for the patient with AML who was going to undergo a stem cell transplant compared to the Wilms’ tumor patient who would not require a transplant as part of their treatment protocol. However, when the same patient was presented with different malnutrition indicators, the nutritional interventions suggested varied greatly. Specifically, when weight loss was presented as a decrease in weight centile as opposed to when the same centiles were presented as z-scores. The reason for this variation in practice is unclear and is outside the questioning asked in this survey. Notably, 68% of respondents reported using weight centiles in their practice while 52% reported using weight-for-length z-scores. It may be that the differences in response reflect respondents answering for a malnutrition indicator that they do not often use in their practice. Despite this, the degree to which the respondents diverged was remarkable (Fig. 3). It may be also that the actual nutritional status of the patient was less impactful in the decision than the perceived intensity of the treatment the patient would be receiving (e.g., chemotherapy vs. transplant) or the type of cancer they had (e.g., Wilms vs. AML) may have affected which interventions respondents chose.

This study has several limitations. First, respondents were limited to members of the MASCC Pediatric Study Group and their institutions. Additionally, many respondents were from the United States and Western Europe. This group may not be reflective of nutritional supportive care globally, especially in low- and middle-income countries. Certain areas globally with resource limitations and higher rates of malnutrition at baseline may merit full screening regardless of diagnosis or expected intensity of treatment. Treatment can be modified accordingly, and several critical papers have outlined several strategies to address nutritional practice approaches taking local culture, resources, and malnutrition baseline rates into context [21, 23]. An additional consideration includes that only 19 respondents completed the survey in its entirety. As such, it should be viewed as exploratory. Furthermore, this highlights that variation in approach may be driven by limitations and variations in formulary, resources, and staffing. It also deserves mentioning that certain indicators and screens may be mandated by local regulatory agencies (e.g., The Joint Commission in the USA), and this can lead to wider variation in nutritional screening at sites as well. Finally, this study was not designed to ascertain causal inference for the variation in care practices described. These limitations notwithstanding, the degree of variability is real, and merits further investigation and attention given the critical nature nutrition plays in children with cancer.

The development of consensus guidelines for nutrition care planning should incorporate such factors to attempt and address nutritional issues more systematically, allowing for patient, disease-specific, and clinical factors to lead to an individualized, yet evidence-based approach to malnutrition. Aiming to decrease variability and better elucidating the factors that ought to be included in such malnutrition assessments and therapies ought to be a priority of future research.

## Conclusions

Three key findings of this exploratory survey of the MASCC Pediatric Study Group include the absence of consistent nutritional screening for pediatric cancer patients, the wide variation in which malnutrition indicators are used, and variability in practice management of malnutrition. The development of consensus recommendations for screening practices, preferred malnutrition indicators, and treatment guidelines could help reduce this variability in care and should be a focus of the global pediatric cancer supportive care community given the critical impact nutrition has on both the short- and long-term outcomes in these vulnerable patients.

## Supplementary Information

Below is the link to the electronic supplementary material.

Supplementary file1 (PDF 115 KB)

## Data Availability

No datasets were generated or analysed during the current study.
