# Supplementary material for: Survey of international pediatric nutritional supportive care practices: a report from the Pediatric Study Group of the Multinational Association of Supportive Care in Cancer (MASCC)
Source: Support Care Cancer. 2024 Sep 7;32(10):644. doi: 10.1007/s00520-024-08826-3 (PMC11380636; doi:10.1007/s00520-024-08826-3)
Supplement: Supplementary file 1 — Supplementary file1 (PDF 115 KB) [file 520_2024_8826_MOESM1_ESM.pdf]

Pediatric Nutrition Practices in Oncology/Stem Cell Transplant

Multinational Association of Supportive Care in Cancer (MASCC) Pediatric Study Group Nutrition Practice Questionnaire

Dear Colleague:

This survey has been developed through an informal committee of the Multinational Association of Supportive Care in Cancer (MASCC) Pediatric Study Group, as a way of forging international pediatric nutrition research collaborations. This questionnaire has been designed to determine international variations of nutrition practice for children and adolescents with cancer and identify key areas for research development.

We are requesting that this questionnaire is completed by you or someone at your institution with more knowledge of the survey content. Eligible people to complete this survey include but are not limited to the following: medical doctors, nurse practitioners, nurses, and registered dietitians. The survey will require about 10-15 minutes to complete.

All data will be kept anonymous, with published results being presented only in aggregate. The activity has been determined to not meet the definition of research, as defined in 45CFR46.102 by the Children's Hospital of Philadelphia Institutional Review Board.

Thank you very much for your participation in this survey. If you have any questions please contact Jason Freedman, Inpatient Medical Director (Oncology/BMT, CHOP) at FreedmanJ@email.chop.edu.

Demographic Information

1. Name:

[ ]

2. Email address:

[ ]

3. Discipline:

- Registered Dietitian (RD)
- Medical Doctor (MD)
- Registered Nurse (RN)
- Certified Registered Nurse Practitioner (CRNP)
- Other (please specify)

Pediatric Nutrition Practices in Oncology/Stem Cell Transplant

Section 2: Institutional Structure

4. Name of institution

5. Where is your institution located? (City/Country)

6. Is your practice center:

- Urban
- Suburban
- Rural

7. What type of medical center is your institution?

- Academic Center
- Commercial (for profit)
- Charity Hospital (not for profit)
- Governmental
- Other (please describe)

8. Does your medical center have an electronic medical record that is used by both physicians and nutritional support staff?

- Yes
- No

9. Approximately how many new diagnoses of pediatric cancer does your center treat annually?

- <50
- 50-100
- 101-200
- 201-400
- >400

10. How many dedicated inpatient oncology beds are at your center?

- 0-15
- 16-30
- 31-45
- 46-60
- >60

11. Approximately many bone marrow transplants (autogeneic and allogeneic) does your center perform annually?

- 0 (My center does not perform bone marrow transplant)
- 1-50
- 51-100
- 101-150
- >150

12a. Does your institution have a dedicated registered dietitian for the inpatient service?

- Yes
- No

12b. If yes, is the RD on rounds?

- Yes
- No

13. What is the full time equivalent (FTE) of a registered dietitian dedicated for your inpatient service? If more than one full time equivalent, please enter the number of full-time equivalents as a number. Examples: 0.5 FTE, 2.25 FTE

14a. Does your institution have a standard patient to registered dietitian ratio (i.e. standard number of inpatients per RD)?

- Yes
- No

14b. If Yes, then enter number of new diagnoses per RD.

15. When does a registered dietitian at your institution see an inpatient oncology patient?

- Every admission
- Every new patient at least once at the time of diagnosis but not every admission
- Every patient at least once (not necessarily at the time of diagnosis) but not every admission
- As needed based on clinical concern
- Other (please specify)

16a. Does your institution use a standardized, validated nutrition screen?

- Yes
- No

16b. If yes, what screen is used?

- Pediatric Nutritional Risk Score (PNRS)
- Subjective Global Nutritional Assessment (SGNA)
- Pediatric Yorkhill Malnutrition Score
- Screening Tool for the Assessment of Malnutrition in Pediatrics (STAMP)
- Screening Tool for Risk of Impaired Nutritional Status and Growth (STRONGkids)
- Other (please specify)

16c. If yes, when is it performed?

- All inpatient admissions
- Inpatient admissions when there is a clinical concern
- All outpatient clinic visits
- Outpatient clinic visits when there is a clinical concern
- Other (please specify)

17a. Does your institution have a standardized guideline for the treatment of undernutrition?

- Yes
- No

17b. If yes, which guidelines do you follow?

- ○ ASPEN
- ○ Royal College of Nursing
- ○ Other

If other, could you share it with us? [insert link to upload]

18. Who performs nutrition consults at your institution?

- ○ My institution does not have a nutrition consult service
- ○ RD
- ○ MD
- ○ Other (please specify)
- ○ RN
- ○ CRNP

19. Which of the following malnutrition indicators are routinely used at your institution for children 1 month to 18 years old? Select all that apply.

- ☐ Weight centile compared to height centile
- ☐ Weight for length z-score
- ☐ Body mass index (BMI) for age z-score
- ☐ Length / height for age z-score
- ☐ Z-score lines in weight for length/height or body mass index for age
- ☐ For children <2 years of age, weight gain velocity relative to the norm
- ☐ Percentage weight for height relative to the ideal
- ☐ Current percentiles for weight (or height) by centiles
- ☐ Mid-upper arm circumference z-scores
- ☐ Weight loss by percent of initial body weight
- ☐ Oral intake by percent of estimated average requirement

Other (please specify)

Pediatric Nutrition Practices in Oncology/Stem Cell Transplant

Section 3: Practice Patterns

Please select the answer that most closely reflects your institutional practice for the

following clinical situations. If your center does not perform stem cell transplants or you are not involved with these patients, please skip to the next section. Thank You!

Subsection A: Treatment at diagnosis

20. New diagnosis of Wilm's tumor, stage 2, as inpatient with normal weight (BMI z-score for age = 0).

- ◯ no nutrition screen is performed and patient does not receive a clinical nutrition consult
- ◯ no nutrition screen is performed and patient receives a clinical nutrition consult
- ◯ nutrition screen is performed and patient does not receive a clinical nutrition consult
- ◯ nutrition screen is performed and patient receives a clinical nutrition consult

21. New diagnosis of Wilm's tumor, stage 2, as inpatient with low weight (BMI z-score for age = -2.2).

- ◯ no nutrition screen is performed and patient does not receive a clinical nutrition consult
- ◯ no nutrition screen is performed and patient receives a clinical nutrition consult
- ◯ nutrition screen is performed and patient does not receive a clinical nutrition consult
- ◯ nutrition screen is performed and patient receives a clinical nutrition consult

22. New diagnosis of High-risk AML with plan for future transplant, as an inpatient with normal weight (BMI z-score for age = 0).

- ◯ no nutrition screen is performed and patient does not receive a clinical nutrition consult
- ◯ no nutrition screen is performed and patient receives a clinical nutrition consult
- ◯ nutrition screen is performed and patient does not receive a clinical nutrition consult
- ◯ nutrition screen is performed and patient receives a clinical nutrition consult

23. New diagnosis of High-risk AML with plan for future transplant, as an inpatient with low weight (BMI z-score for age = -2.2).

- ◯ no nutrition screen is performed and patient does not receive a clinical nutrition consult
- ◯ no nutrition screen is performed and patient receives a clinical nutrition consult
- ◯ nutrition screen is performed and patient does not receive a clinical nutrition consult
- ◯ nutrition screen is performed and patient receives a clinical nutrition consult

Pediatric Nutrition Practices in Oncology/Stem Cell Transplant

Section 3: Practice Patterns

Please select the answer that most closely reflects your institutional practice for the following clinical situations.

Subsection B: Peri-bone marrow transplant treatment

24. Relapsed patient with AML admitted as an inpatient for allogeneic match-sibling bone marrow transplant with normal weight (BMI z-score for age = 0).

- ◯ no nutrition screen is performed and patient does not receive a clinical nutrition consult
- ◯ no nutrition screen is performed and patient receives a clinical nutrition consult
- ◯ nutrition screen is performed and patient does not receive a clinical nutrition consult
- ◯ nutrition screen is performed and patient receives a clinical nutrition consult

25. Relapsed patient with AML admitted as an inpatient for allogeneic match-sibling bone marrow transplant with low weight (BMI z-score for age = -2.2).

- ◯ no nutrition screen is performed and patient does not receive a clinical nutrition consult
- ◯ no nutrition screen is performed and patient receives a clinical nutrition consult
- ◯ nutrition screen is performed and patient does not receive a clinical nutrition consult
- ◯ nutrition screen is performed and patient receives a clinical nutrition consult

26a. Relapsed patient with AML admitted as an inpatient for allogeneic match-sibling bone marrow transplant with normal weight. What is your standard practice for nutrition support during a transplant admission?

- ☐ provide additional calorie support enterally or parenterally as needed if malnutrition indicators are met
- ☐ begin enteral supplementation with NG feeds on a standardized day (e.g. Day -1)
- ☐ begin parenteral supplementation with TPN on a standardized day (e.g. Day +5)

If yes, what day?

____

27a. Relapsed patient with AML admitted as an inpatient for allogeneic match-sibling bone marrow transplant with low weight. What is your standard practice for nutrition support during a transplant admission?

- ☐ provide additional calorie support enterally or parenterally as needed
- ☐ begin enteral supplementation with NG feeds on a standardized day (e.g. Day -1)
- ☐ begin parenteral supplementation with TPN on a standardized day (e.g. Day +5)

If yes, what day?

____

Section 3: Practice Patterns

Please select the answer that most closely reflects your institutional practice for the following clinical situations.

Subsection C: Nutritional management during treatment

In which of the following inpatient situations would a typical provider at your institution place a consult to clinical nutrition? In each scenario the admission is for fever and neutropenia in an otherwise well-appearing patient. Select Yes or No.

☐ If your institution formally consults clinical nutrition for 100% of admissions and never deviates from this practice please check here.

☐ If your institution does not have a clinical nutrition consult service available for your patients please check here.

28. After 2 cycles of therapy on DD-4A (vincristine, dactinomycin, and doxorubicin), a patient with Wilm’s tumor (Stage 2), has lost 3% of his weight compared to diagnosis.

○ Yes

○ No

29. After 2 cycles of therapy on DD-4A (vincristine, dactinomycin, and doxorubicin), a patient with Wilm’s tumor (Stage 2), has lost 6% of his weight compared to diagnosis.

○ Yes

○ No

30. After 2 cycles of therapy on DD-4A (vincristine, dactinomycin, and doxorubicin), a patient with Wilm’s tumor (Stage 2), has lost 12% of his weight compared to diagnosis.

○ Yes

○ No

31. At the end of consolidation, a patient with standard risk B-cell ALL has lost 3% of his weight compared to diagnosis.

○ Yes

○ No

32. At the end of consolidation, a patient with standard risk B-cell ALL has lost 6% of his weight compared to diagnosis.

○ Yes

○ No

33. At the end of consolidation, a patient with standard risk B-cell ALL has lost 12% of his weight compared to diagnosis.

○ Yes

○ No

34. At the end of induction II, a patient with High risk AML with plan for future transplant, has lost 3% of his body weight compared to diagnosis.

○ Yes

○ No

35. At the end of induction II, a patient with High risk AML with plan for future transplant, has lost 6% of his body weight compared to diagnosis.

- Yes
- No

36. At the end of induction II, a patient with High risk AML with plan for future transplant, has lost 12% of his body weight compared to diagnosis.

- Yes
- No

Pediatric Nutrition Practices in Oncology/Stem Cell Transplant

Subsection C: Nutritional management during treatment

For the following questions, please select the most likely treatment recommendation at your institution for the patients described in the question stem. In each scenario the patient is admitted to the inpatient oncology unit for fever and neutropenia and is otherwise well-appearing.

37. After 2 cycles of therapy on DD-4A (vincristine, dactinomycin, and doxorubicin), a patient with Wilm's tumor (Stage 2), has lost 3% of his weight compared to diagnosis.

- no intervention needed
- start oral supplement
- start appetite stimulant
- place NGT and start enteral supplement
- start TPN

38. After 2 cycles of therapy on DD-4A (vincristine, dactinomycin, and doxorubicin), a patient with Wilm's tumor (Stage 2), has lost 6% of his weight compared to diagnosis.

- no intervention needed
- start oral supplement
- start appetite stimulant
- place NGT and start enteral supplement
- start TPN

39. After 2 cycles of therapy on DD-4A (vincristine, dactinomycin, and doxorubicin), a patient with Wilm's tumor (Stage 2), has lost 12% of his weight compared to diagnosis.

- no intervention needed
- start oral supplement
- start appetite stimulant
- place NGT and start enteral supplement
- start TPN

40. At the end of consolidation, a patient with standard risk B-cell ALL has lost 3% of his weight compared to diagnosis.

- ◯ no intervention needed
- ◯ place NGT and start enteral supplement
- ◯ start oral supplement
- ◯ start TPN
- ◯ start appetite stimulant

41. At the end of consolidation, a patient with standard risk B-cell ALL has lost 6% of his weight compared to diagnosis.

- ◯ no intervention needed
- ◯ place NGT and start enteral supplement
- ◯ start oral supplement
- ◯ start TPN
- ◯ start appetite stimulant

42. At the end of consolidation, a patient with standard risk B-cell ALL has lost 12% of his weight compared to diagnosis.

- ◯ no intervention needed
- ◯ place NGT and start enteral supplement
- ◯ start oral supplement
- ◯ start TPN
- ◯ start appetite stimulant

43. At the end of induction II, a patient with High risk AML with plan for future transplant, has lost 3% of his body weight compared to diagnosis.

- ◯ no intervention needed
- ◯ place NGT and start enteral supplement
- ◯ start oral supplement
- ◯ start TPN
- ◯ start appetite stimulant

44. At the end of induction II, a patient with High risk AML with plan for future transplant, has lost 6% of his body weight compared to diagnosis.

- ◯ no intervention needed
- ◯ place NGT and start enteral supplement
- ◯ start oral supplement
- ◯ start TPN
- ◯ start appetite stimulant

45. At the end of induction II, a patient with High risk AML with plan for future transplant, has lost 12% of his body weight compared to diagnosis.

- ◯ no intervention needed
- ◯ place NGT and start enteral supplement
- ◯ start oral supplement
- ◯ start TPN
- ◯ start appetite stimulant

Pediatric Nutrition Practices in Oncology/Stem Cell Transplant

Subsection C: Nutritional management during treatment

For the following questions, please select the most likely treatment recommendation at your institution. In each scenario the patient is admitted to the inpatient oncology unit and is otherwise well-appearing.

46. A patient with high risk B-cell ALL is admitted to the oncology unit for high dose methotrexate at the beginning of interim maintenance I. On admission it is noted that the weight centile is 60% and the height centile is 75%.

- ◯ no intervention needed
- ◯ place NGT and start enteral supplement
- ◯ start oral supplement
- ◯ start TPN
- ◯ start appetite stimulant

47. A patient with high risk B-cell ALL is admitted to the oncology unit for high dose methotrexate at the beginning of interim maintenance I. On admission it is noted that the weight centile is 50% and the height centile is 75%.

- ◯ no intervention needed
- ◯ place NGT and start enteral supplement
- ◯ start oral supplement
- ◯ start TPN
- ◯ start appetite stimulant

48. A patient with high risk B-cell ALL is admitted to the oncology unit for high dose methotrexate at the beginning of interim maintenance I. On admission it is noted that the percentage weight for height it is 92% of the ideal.

- ◯ no intervention needed
- ◯ place NGT and start enteral supplement
- ◯ start oral supplement
- ◯ start TPN
- ◯ start appetite stimulant

49. A patient with high risk B-cell ALL is admitted to the oncology unit for high dose methotrexate at the beginning of interim maintenance I. On admission it is noted that the percentage weight for height it is 85% of the ideal.

- ◯ no intervention needed
- ◯ place NGT and start enteral supplement
- ◯ start oral supplement
- ◯ start TPN
- ◯ start appetite stimulant

50. A patient with high risk B-cell ALL is admitted to the oncology unit for high dose methotrexate at the beginning of interim maintenance I. On admission it is noted that the weight for length z-score is -0.8.

- ◯ no intervention needed
- ◯ place NGT and start enteral supplement
- ◯ start oral supplement
- ◯ start TPN
- ◯ start appetite stimulant

51. A patient with high risk B-cell ALL is admitted to the oncology unit for high dose methotrexate at the beginning of interim maintenance I. On admission it is noted that the weight for length z-score is -1.2.

- no intervention needed
- start oral supplement
- start appetite stimulant
- place NGT and start enteral supplement
- start TPN

52. A patient with high risk B-cell ALL is admitted to the oncology unit for high dose methotrexate at the beginning of interim maintenance I. On admission it is noted that the weight for length z-score is -2.2.

- no intervention needed
- start oral supplement
- start appetite stimulant
- place NGT and start enteral supplement
- start TPN

53. A patient with high risk B-cell ALL is admitted to the oncology unit for high dose methotrexate at the beginning of interim maintenance I. On admission it is noted that the weight for length z-score is -3.2.

- no intervention needed
- start oral supplement
- start appetite stimulant
- place NGT and start enteral supplement
- start TPN

54. A patient with high risk B-cell ALL is admitted to the oncology unit for high dose methotrexate at the beginning of interim maintenance I. On admission it is noted that the weight centile has decreased from the 85th centile to the 70th centile.

- no intervention needed
- start oral supplement
- start appetite stimulant
- place NGT and start enteral supplement
- start TPN

55. A patient with high risk B-cell ALL is admitted to the oncology unit for high dose methotrexate at the beginning of interim maintenance I. On admission it is noted that the weight centile has decreased from the 85th centile to the 60th centile.

- no intervention needed
- start oral supplement
- start appetite stimulant
- place NGT and start enteral supplement
- start TPN

56. A patient with high risk B-cell ALL is admitted to the oncology unit for high dose methotrexate at the beginning of interim maintenance I. On admission it is noted that the weight centile has decreased from the 86th centile to the 46th centile.

- no intervention needed
- start oral supplement
- start appetite stimulant
- place NGT and start enteral supplement
- start TPN

57. A patient with high risk B-cell ALL is admitted to the oncology unit for high dose methotrexate at the beginning of interim maintenance I. On admission it is noted that the weight centile has decreased from the 99th centile to the 82nd centile.

- no intervention needed
- place NGT and start enteral supplement
- start oral supplement
- start TPN
- start appetite stimulant

58. A patient with high risk B-cell ALL is admitted to the oncology unit for high dose methotrexate at the beginning of interim maintenance I. On admission it is noted that the weight for length z-score has decreased from 1.1 to -0.1.

- no intervention needed
- place NGT and start enteral supplement
- start oral supplement
- start TPN
- start appetite stimulant

59. A patient with high risk B-cell ALL is admitted to the oncology unit for high dose methotrexate at the beginning of interim maintenance I. On admission it is noted that the weight for length z-score has decreased from 2.1 to 0.9.

- no intervention needed
- place NGT and start enteral supplement
- start oral supplement
- start TPN
- start appetite stimulant

60. A patient with high risk B-cell ALL is admitted to the oncology unit for high dose methotrexate at the beginning of interim maintenance I. On admission it is noted that over the past week, the patient's oral intake is providing 80% of the estimated energy needs.

- no intervention needed
- place NGT and start enteral supplement
- start oral supplement
- start TPN
- start appetite stimulant

61. A patient with high risk B-cell ALL is admitted to the oncology unit for high dose methotrexate at the beginning of interim maintenance I. On admission it is noted that over the past week, the patient's oral intake is providing 73% of the estimated energy needs.

- no intervention needed
- place NGT and start enteral supplement
- start oral supplement
- start TPN
- start appetite stimulant

62. A patient with high risk B-cell ALL is admitted to the oncology unit for high dose methotrexate at the beginning of interim maintenance I. On admission it is noted that over the past week, the patient's oral intake is providing 60% of the estimated energy needs.

- no intervention needed
- place NGT and start enteral supplement
- start oral supplement
- start TPN
- start appetite stimulant

63. A patient with high risk B-cell ALL is admitted to the oncology unit for high dose methotrexate at the beginning of interim maintenance I. On admission it is noted that over the past week, the patient’s oral intake is providing 20% of the estimated energy needs.

- ○ no intervention needed
- ○ start oral supplement
- ○ start appetite stimulant
- ○ place NGT and start enteral supplement
- ○ start TPN
